# Memoirs on a Few Fundamental Points of Dental Medicine, Considered in Its Application to Hygiene and Therapeutics

**Published:** 1854-07

**Authors:** A. F. Talma, C. A. Du Bouchet

**Affiliations:** Dentist to the King of Belgium, &c.


### ARTICLE XVIII.

*Memoirs on a Few Fundamental Points of Dental Medicine, considered in its Application to Hygiene and Therapeutics.* By A. F. TALMA, M. D., Dentist to the King of Belgium, &c. Translated by C. A. DU BOUCHET, M. D., D. D. S. First Series. Brussels, 1852.

*Cares relating to Second Dentition.*—The defective arrangements of teeth are usually limited to incisors and canines. It is more rare to find bicuspids out of range. As regards molars, although assuming almost always an internal oblique direction, they straighten as the alveolar borders enlarge, and also under the influence of the eccentric pressure acting upon them.

As a general thing, the irregularity of one tooth causes that of several others in the same arch, and likewise in the opposite jaw. Thus, an incisor inverted or everted, or merely twisted on its axis, will cause its neighbor to incline towards it in an opposite position, and thus the deformity may affect all the front teeth. On the other part, the corresponding teeth of the opposite jaw being subjected to abnormal pressure during mastication, and the occlusion of the mouth, depart almost necessarily, from their regular position. From this *ensemble* of influences result combinations varying ad infinitum, all assuming the type of more or less shocking deformities, interfering with the due performance of functions and predisposing to the premature loss of the very teeth themselves.

Among the cares to be bestowed, during the second dentition, upon the arrangement of permanent teeth, the extraction of deciduous or milk teeth is that which taxes most parents and dentists. It then appears obvious that, in order that the tooth of replacement may have room to assume its proper position, that nothing better could be done than to prepare that room in advance. But reason, and especially experience, modifying much this too hasty conclusion, two difficulties must at this juncture be carefully guarded against. The one consists in too early an extraction of the deciduous teeth, the other, in allowing them to remain beyond the necessary period.*

Experience and a considerate appreciation of the conditions of the dental system, can alone prevent the errors I have just pointed out, and fix the truly proper time for operating. I have ascertained that more frequent and serious irregularities are caused by too early extraction, than by the opposite course. The deformities produced on account of having allowed deciduous teeth to remain too long a time in their situation, very frequently correct themselves, from the very growth and enlargement of the alveolar arch and of the whole maxillary. We should never forget that the crowns of teeth after their eruption no longer increase in size, while the bone which supports them remains for several years subject to the laws of development of the entire system. Whence there results—that at the end of a certain time, some teeth which appeared never to be able to find sufficient room, finally arrange themselves in the most perfect order, much to the astonishment of parents and often dentists themselves. When, in the cases, which I believe more scarce and resulting from special dispositions, the deformity is more considerable and appears to have a tendency to become permanent, we can still avert it by such operations as I shall point out subsequently. We shall find that in almost every case we can remedy the difficulty, while the exaggerated separation of the teeth, or the loss of those which have been sacrificed, ordinary results of an opposite practice, usually leave no resource.

As a consequence of these principles, deciduous molars, even carious and painful, should not be extracted before the age of eight or nine, unless, in consequence of this condition of things, the health of the child should be positively disturbed. Heretofore, I have much applauded myself for temporizing, and

*We omit here the enumeration of cases illustrating the views of the author, in regard to the proper indications for extracting deciduous teeth, as they are fully summed up in the paragraphs which will follow. D. B.

having recourse to palliatives, local applications, sometimes plugging, or even cauterization. When the crown of those teeth has at last yielded to decay and has gradually broken entirely off, I still deem it useful to respect their roots. Their premature extraction would allow the first permanent molars appearing from the sixth to the seventh year, to encroach upon the room they occupy; so that the bicuspids in their turn should, in order to find room, crowd forward as far as the lateral incisors, thus excluding the canine teeth from the circle. In such cases canine teeth have often been sacrificed by too thoughtless practitioners; such mutilations should ever be avoided. We may indeed be at times compelled to extract a sound permanent tooth, but let not that be a canine, let us choose the bicuspid—the canine tooth will soon assume its proper place.

To sum up, a long practice has demonstrated to me that the dentist, as far as regards operations to be performed upon deciduous teeth, should observe the following rules as the most rational and the safest.

1st. So long as deciduous teeth remain firm, even when they begin to become loose, if no indication heralds the near apparition of the permanent teeth, nature should be abandoned to itself; there is no call for interference. The dentist is authorized to extract a deciduous tooth only when it has become so loose as to seem ready to drop out, and when a slight effort is sufficient to detach it.

2d. When in front of, or behind a milk tooth still firm or already loosened, appears a circumscribed reddish, somewhat painful tumefaction, under which a projecting hard body may sometimes be felt, it is proper to extract that tooth. That is the best means to prevent the deviation of the tooth endeavoring to come out. Consequently, if that tooth has already pierced the gum, we should perform the extraction so as to allow it to fall in the line before the deviation becomes more considerable, presents more difficulty to be corrected.

3d. If one of the permanent teeth does not find in the space left by the deciduous one, room enough to locate itself properly, and in consequence of want of room begins to deviate, we may extract the contiguous milk tooth. But, far from hurrying to perform this operation, it should be retarded until the necessity for it be clearly demonstrated, and generally until one-half of the crown of the new tooth has pushed through the gum. It is only at this time that this tooth has exercised all its expansive force, that its position is certain, and that the removal of the contiguous deciduous tooth can be but advantageous.

These operations should follow the progress of the eruption of the teeth, beginning at the central incisors, and following with the lateral incisors and canines.

4th. In certain cases, after having sacrificed, accordingly with that which precede the deciduous canine to make room for the permanent lateral incisor, we may be obliged still to extract the first bicuspid which appears at nine years, to make room for the canine which comes at eleven. But it sometimes happens in opposition to the general rule, that the canine tooth erupts before the bicuspid; we should in this case extract the first deciduous molar. These operations should be performed as soon as the necessity for them is ascertained, because, as I have already stated, we have nothing to gain by waiting, and also because the extraction of those teeth is the easier as their roots are less fully developed.

5th. We meet, rather frequently, with children whose canines are well set but have appeared before the first bicuspid; in such cases the lateral incisor not finding room enough in the arch, deviates anteriorly or posteriorly; its extraction then becomes indispensable. The ulterior arrangement may be left to nature, which will direct the other teeth towards the vacancy, and thus fill up the breach.

During the whole period of life,* teeth have a manifest tendency to progress forward; and this tendency may, in many circumstances, be turned to good account, in regulating their arrangement. Especially, when a case similar to that which we have just mentioned is met with in the lower maxillary, my

* This fact seems to us well worthy to be borne in mind, as in many cases great advantage may be derived from it. D. B.

rule is always to extract the most anterior incisor, even if properly set. Its removal is less difficult and painful than that of the other, which, by the action of the tongue or with the aid of some of the arrangements mentioned in the following memoir, will assume the place of the extracted one.

6th. The final period of dentition, the eruption of the last permanent molars or dentes sapientiæ, frequently calls the attention of the dentist, and claims his assistance. It sometimes happens, especially in subjects who have passed the normal epoch for the cutting of those teeth, that their place being invaded by the second molars, they find no room to arrange themselves. This obstacle is more frequent in the inferior than in the upper maxillary, on account of the coronoid apophysis forming at the extremity of the arch or bony boundary, which will not yield. Held back within the thickness of the bone, and endeavoring to escape from their place of concealment, the wisdom teeth under such circumstances are apt to cause dull, deep-seated pains radiating over the entire side of the dental arch, reaching the auricular and temporal regions and even extending to the whole side of the head and face, keeping up a continual state of suffering and irritation, ultimately felt by the general system and impairing the health of the patient. Almost invariably this state of things is attributed to any but the real cause, and the physician himself, when called in, frequently neglects the examination of parts, and is prone to attribute to the general and confused influences of cold, dampness, rheumatic tendency, and various neuralgic affections, the origin and persistency of the morbid phenomena presented.

The principal, and we may say, the only difficulty in such a case consists in making a correct diagnosis. Ascertaining the number of teeth, the original seat of pain, its vague character, its irregular nocturnal exacerbations, are so many circumstances which will assist the dentist. If, upon touching the gum behind the second molar, we find a particularly painful spot and considerable swelling, the gum should be deeply incised, and a probe inserted in the wound to feel the crown of the tooth endeavoring to cut its way through. The subsequent treatment must vary as either of the three following cases shall occur.

If the tooth seems to have room for its development and has only been retarded by the toughness or the thickness of the integuments, the exploring incision should be enlarged, made crucial, a fragment of the gum over it may be cut out—nothing further need be done—time will effect the cure.

If the wisdom tooth cannot be accommodated between the second molar and the coronoid apophysis, it must be extracted if accessibe to instruments.

Finally, if the wisdom tooth is not accessible to instruments, the second molar must be extracted; its place will soon be filled up by the new tooth. Generally, rational practice is also here preservative practice, to save both teeth if their position permits; in the contrary case, prefer to remove the dentes sapientiæ, which, more frequently than the second molar, is liable to caries, and always less solid and less useful to mastication; such is the rule to be followed.

I cannot repeat too often a counsel justified by the preceding remarks: the mouth of children should be, during the period of second dentition, examined regularly at suitable intervals by a skillful dentist, who alone can appreciate the progress of dental eruption and rectify its irregularities at the proper time.—*Dental News Letter.*

